# Supplementary material for: Selenomethionine Antagonized microRNAs Involved in Apoptosis of Rat Articular Cartilage Induced by T-2 Toxin
Source: Toxins (Basel). 2023 Aug 4;15(8):496. doi: 10.3390/toxins15080496 (PMC10467099; doi:10.3390/toxins15080496)
Supplement: Supplementary file 1 [file toxins-15-00496-s001.zip › toxins-2514703-supplementary.pdf]

Supplementary Materials: Selenomethionine Antagonized microRNAs Involved in Apoptosis of Rat Articular Cartilage Induced by T-2 Toxin

Fangfang Yu, Kangting Luo, Miao Wang, Jincai Luo, Lei Sun, Shuiyuan Yu, Juan Zuo and Yanjie Wang

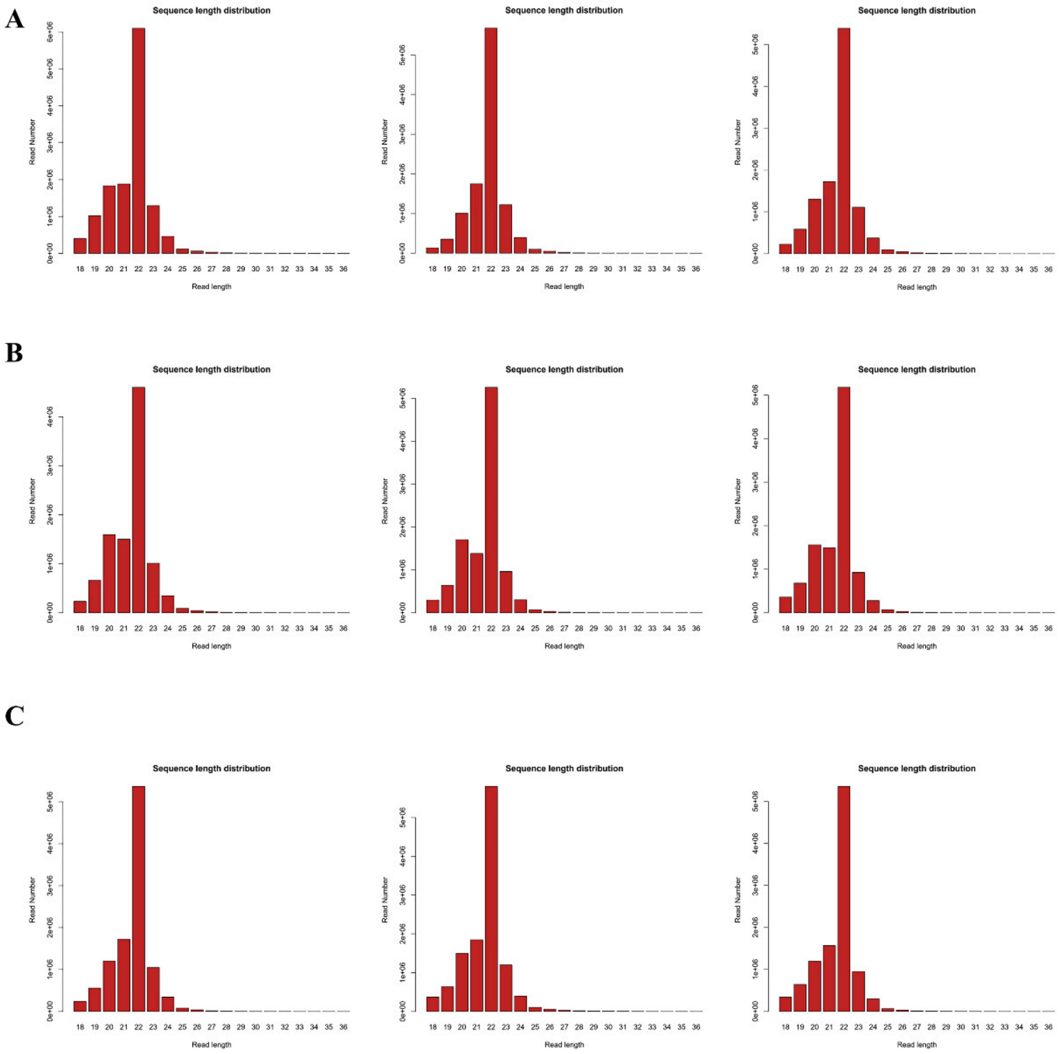

Figure S1. The length of the small RNA. A, B, C represents the length of small RNA of three parallel samples in the control group, T-2 group, and T-2 + SeMet group, respectively. The x-coordinate represents the length of reads, and the y-coordinate represents the number of reads of corresponding length.

Table S1. The primer sequences used for RT-qPCR.

miRNA	Forward (5′-3′)	Reverse (5′-3′)
miR-206-3p	GCGCGTGGAATGTAAGGAAGT	GTCGTATCCAGTGCAGGGTCCGAGGTATTCGCACTGGATACGACCCACAC
miR-204-5p	GGCGTTCCCTTTGTCATCCT	GTCGTATCCAGTGCAGGGTCCGAGGTATTCGCACTGGATACGACAGGCAT
miR-376c-5p	GGGCGGTGGATATTCCTTCT	GTCGTATCCAGTGCAGGGTCCGAGGTATTCGCACTGGATACGACAAACAT
